# High Diversity at PRDM9 in Chimpanzees and Bonobos

**DOI:** 10.1371/journal.pone.0039064

**Published:** 2012-07-02

**Authors:** Linn Fenna Groeneveld, Rebeca Atencia, Rosa M. Garriga, Linda Vigilant

**Affiliations:** 1 Department of Primatology, Max Planck Institute for Evolutionary Anthropology, Leipzig, Germany; 2 Réserve Naturelle Sanctuaire à Chimpanzés de Tchimpounga, Jane Goodall Institute, Pointe-Noire, Republic of Congo; 3 Tacugama Chimpanzee Sanctuary, Freetown, Sierra Leone; Aarhus University, Denmark

## Abstract

**Background:**

The PRDM9 locus in mammals has increasingly attracted research attention due to its role in mediating chromosomal recombination and possible involvement in hybrid sterility and hence speciation processes. The aim of this study was to characterize sequence variation at the PRDM9 locus in a sample of our closest living relatives, the chimpanzees and bonobos.

**Methodology/Principal Findings:**

PRDM9 contains a highly variable and repetitive zinc finger array. We amplified this domain using long-range PCR and determined the DNA sequences using conventional Sanger sequencing. From 17 chimpanzees representing three subspecies and five bonobos we obtained a total of 12 alleles differing at the nucleotide level. Based on a data set consisting of our data and recently published *Pan* PRDM9 sequences, we found that at the subspecies level, diversity levels did not differ among chimpanzee subspecies or between chimpanzee subspecies and bonobos. In contrast, the sample of chimpanzees harbors significantly more diversity at PRDM9 than samples of humans. *Pan* PRDM9 shows signs of rapid evolution including no alleles or ZnFs in common with humans as well as signals of positive selection in the residues responsible for DNA binding.

**Conclusions and Significance:**

The high number of alleles specific to the genus *Pan*, signs of positive selection in the DNA binding residues, and reported lack of conservation of recombination hotspots between chimpanzees and humans suggest that PRDM9 could be active in hotspot recruitment in the genus *Pan*. Chimpanzees and bonobos are considered separate species and do not have overlapping ranges in the wild, making the presence of shared alleles at the amino acid level between the chimpanzee and bonobo species interesting in view of the hypothesis that PRDM9 plays a universal role in interspecific hybrid sterility.

## Introduction

In sexually reproducing organisms, meiotic recombination is a crucial process during which crossover events during the first meiotic division ensure the correct alignment and segregation of homologous chromosomes. This produces new allelic combinations via the breaking and reforming of double strands, and thus yields new genetic diversity upon which selection can act. Such double-strand breaks do not occur randomly throughout the genome, but instead are clustered spatially in 1–2 kb long stretches, termed hotspots [1]–[4].

A number of research groups recently identified PRDM9 as a gene involved in the specification of hotspots in mice and humans [5]–[7]. Intriguingly, PRDM9 was also identified as the identity of a hybrid sterility locus in mice that was first described over 35 years ago [8]. Accordingly, as a result of its apparently important role in recombination and potentially significant role in the speciation process, this gene has become the focus of intense study and interest [9]–[15].

The PRDM9 locus contains an N-terminal KRAB, SSXRD and a PR/SET domain, followed by a variably long C-terminal zinc finger (ZnF) array [16]–[18]. In contrast to the other domains, the DNA binding domain (ZnF array) of PRDM9 was found to be evolving rapidly in rodents and across the primate lineage and the residues responsible for DNA binding show signs of positive selection in these taxa. Furthermore, single zinc finger sequences within each ZnF array are more similar to one another than to zinc fingers of the array in closely related species, suggesting concerted evolution within the arrays [7], [19], [20]. One particular 13 mer DNA motif is associated with roughly 40% of human hotspots [21] but is not active in chimpanzees, and observed patterns of motif evolution suggest that the motif was activated along the human lineage, as opposed to inactivated in chimpanzees [7]. One possible explanation for the rapid evolution of the zinc finger array is that the inevitable destruction of the hotspot motifs through recombination itself (hotspot conversion paradox) is counterbalanced by selection for novel binding targets [14], [15], [22], [23].

Allelic diversity of the PRDM9 ZnF domain has been characterized in humans and more than 40 alleles with 8–19 ZnF repeats have been identified to date [5], [6], [19], [24]–[26]. One allele (A) has been found to be present at high frequency overall and especially in populations of mainly non-African ancestry (85%), but at a lower frequency in African ancestry populations (47%). Most of the alleles identified in addition to allele A occur at much lower frequencies (under 5%, data from [6], [19], [24], [26]). Studies of recombination patterns, combined with PRDM9 allele-typing, suggest that allelic variation in PRDM9 accounts for almost all of the variation in hostpot activity [6], [24]–[28]. Specifically, allele A, which is predominant among Europeans, binds *in vitro* to the degenerate 13 mer motif found in historic recombination hotspots identified in Europeans [6], [7], whereas another allele (I) was shown to preferentially binds its own predicted motif [6]. Thus, although it seems clear that allelic variation in PRDM9 has a pronounced effect on recombination patterns, it still remains a puzzle how dominance effects in heterozygous individuals affect recombination (summarized in [15]), why the genetic background, on which the 13 mer motif is found, seems to influence the likelihood that the motif is associated with a hotspot [15], [21] and why activation of a hotspot is not predictably dependent on the presence of a specific binding motif [26]. Furthermore, it is still unclear to date how universal a role PRDM9 has in causing hybrid sterility (e.g. [14], [15]).

A hybrid sterility gene, that due to a Muller-Dobzhanzky incompatibility leads to sterility of the male F_1_ in some crosses of *Mus mus musculus* and *M. m. domesticus,* was first described over three decades ago [29], [30] and subsequently identified as PRDM9 [8]. Depending on the specific mouse strain, some *M. m. musculus* and *M. m. domesticus* hybrids exhibit spermatogenetic failure depending on the PRDM9 alleles (previously termed Hst1^s^ (sterile) and Hst1^f^ (fertile)) and the origin of the X chromosome involved [8], [31]. Moreover, a human study examining infertile and fertile Japanese men found that three SNPs which alter DNA binding residues of the ZnF array were found significantly more often in the proven-fertile group [32]. This, together with PRDM9 being present and apparently under positive selection in many taxa, leads to the suggestion that variation in PRDM9 could be involved in hybrid sterility in a number of species [13]–[15]. More specifically, it has been hypothesized that reproductively isolated species should be distinguishable by their PRDM9 alleles, if this gene plays a universal role in hybrid sterility [14].

Although the domain architecture of PRDM9 is generally conserved across metazoans [17], [18], the gene is lacking in some taxa, such as in chickens (*Gallus gallus*), frog (*Xenopus tropicalis*) and fruit fly (*Drosophila melanogaster*) and apparently non-functional in others, such as opossum (*Monodelphis domestica*), nematode (*Caenorhabditis elegans*) and dog [19], [33], [34]. Moreover, the gene is present in some taxa, such as ray-finned fishes (i.e. *Danio rerio*) and tunicates (i.e. *Ciona intestinalis*), but characteristic features, such as signals of positive selection and/or concerted evolution, are lacking, thus suggesting that in these taxa PRDM9 is not active in recombination hotspot regulation [19]. It is thus not clear to what an extent PRDM9 is universally active in hotspot regulation across metazoans.

To date, allelic diversity of PRDM9 has only been well characterized in humans [6], [24]–[28] and to a lesser extent in mice [6], [35] and western chimpanzees [36]. The data available for all other species is either based on sequencing of PRDM9 in single individuals (rodents: [19]) or on genome data [20], which depending on the sequencing and assembly methods employed may be unreliable, due to the challenge posed by the highly repetitive structure of the locus. Characterization of PRDM9 variation in other species beyond humans, western chimpanzees and mice is paramount for addressing outstanding questions about the function and evolution of PRDM9. As a first step towards this goal, we sequenced the C-terminal ZnF array in representatives of three chimpanzee subspecies and bonobos in order to characterize allelic diversity of PRDM9 in our closest living relatives.

## Results

**Figure 1:**
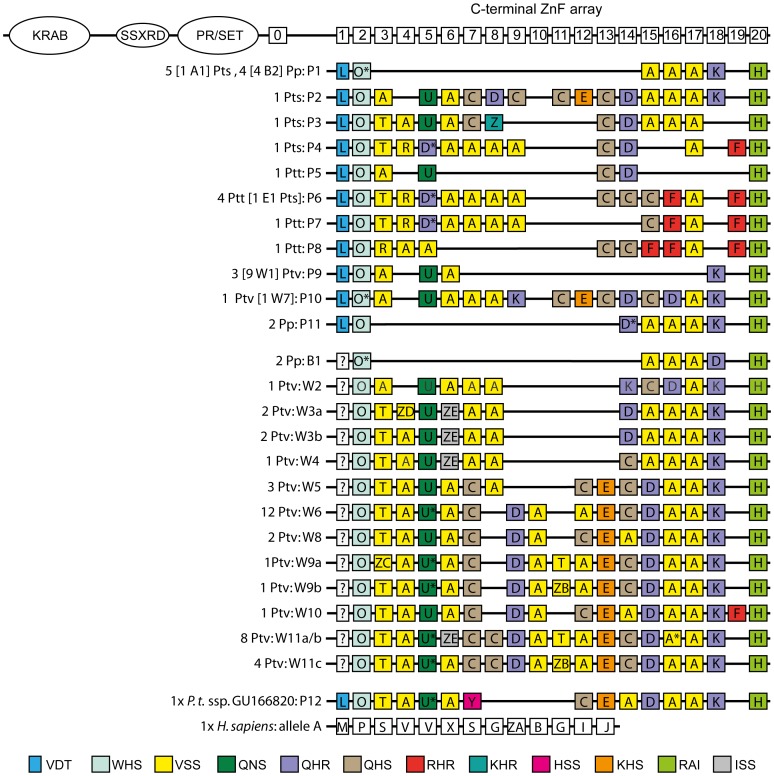
Schematic representation of PRDM9 domains and allelic variation in *Pan*. The top block depicts alleles identified in this study. The second block shows the additional alleles characterized by Auton et al. [36]. The four alleles common to both studies are shown in the top block, with the number of occurrences and the corresponding (sub-)species given in square brackets. Pp  =  *Pan paniscus,* Ptv  =  *P. troglodytes verus*, Ptt  =  *P. t. troglodytes*, Pts  =  *P. t. schweinfurthii*. Different ZnF repeats are coded by letters and repeats marked with a * differ from those with the same letter code by one, two, or three synonymous substitutions. The underlying nucleotide sequence, as shown in Figure 2, of O* is n or zg, D* represents q, A* is zf and U* represents w. Colors correspond to the AA residue combination at positions −1, 3 and 6 of the ZnFs, as given in the legend. Residue position 2, which also plays a role in DNA binding is fixed (serine) and therefore not shown. Human allele A is depicted for reference.

We sequenced the C-terminal zinc finger (ZnF) array of the last exon of the PRDM9 gene, excluding the first ZnF repeat because it lies outside the array (“0” in schematic representation of PRDM9 in Fig. 1). The first ZnF repeat within the actual array (“1” in Fig. 1) is truncated and thus was not included in all analyses (see Methods). We obtained 25 PRDM9 DNA sequences from 17 chimpanzees and five bonobos. Ten of the 22 individuals may possibly be heterozygous, but only in three cases was it possible to identify the second allele (see Methods and Table S1). Thus, it is possible that we underestimate the diversity at PRDM9 in this sample of chimpanzees and bonobos. In total, twelve alleles differing at the nucleotide level were identified in the 22 *Pan* individuals, which corresponds to 11 alleles at the amino acid level.

**Figure 2:**
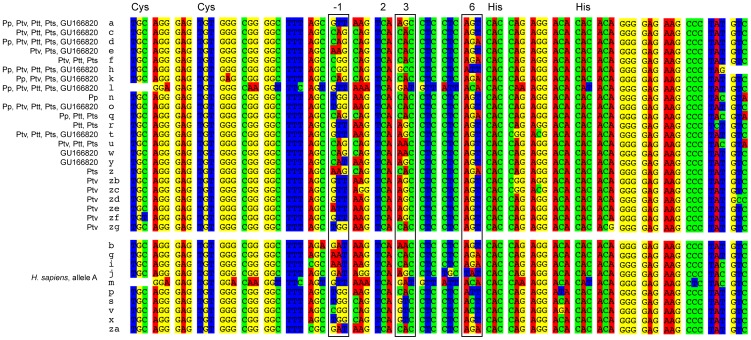
Alignment of PRDM9 ZnF repeats of 52 *Pan* individuals and one human. The ZnF repeats identified in 82 *Pan* alleles of which 28 are unique DNA sequences, including data from Auton et al. [36] and Oliver at al. (GU166820: [19]), are depicted in the top block. Pp  =  *Pan paniscus,* Ptv  =  *P. troglodytes verus*, Ptt  =  *P. t. troglodytes*, Pts  =  *P. t. schweinfurthii*. The second block depicts the ZnF repeats of the human A allele for comparison with those identified in *Pan*. For comparative purposes, we adhere to the break between repeats chosen by Oliver et al. [19]. The two conserved cysteine and histidine residues are marked at the top and positions −1, 3 and 6 of the alpha helices are identified by black frames.

The DNA sequences we obtained contain seven to 17 ZnF repeats (Fig. 1). There are 14 polymorphic sites in an alignment of all *Pan* ZnF repeats, excluding the slightly aberrant first repeat. Half of these polymorphic sites are found at residues −1, 3 and 6 of the ZnF α-helixes, which are the sites suggested to be responsible for the DNA-binding specificity of PRDM9 (Fig. 2, e.g. [37]). There are no shared ZnF repeats between the published human PRDM9 sequences and those identified in the genus *Pan.*

For comparison we also include in Figure 1 the results from a recent study in which 56 DNA sequences were found in a sample of 25 western chimpanzees, 1 eastern chimpanzee and 3 bonobos. These represented 19 alleles differing at the nucleotide level and translate into 17 alleles differing at the amino acid level (Fig. 1) [36]. Of these 17 amino acid sequences identified by Auton et al. [36], four are also present in our data set.

### Diversity Among *Pan* Species and Subspecies

In a combined data set comprised of our data and that from a recent study [36], a total of 81 PRDM9 DNA sequences were obtained from a total of 51 *Pan* individuals (29 western, 6 central, 8 eastern chimpanzees and 8 bonobos) and 27 unique PRDM9 DNA sequences were observed. Of these, 24 were found in chimpanzees and 3 in bonobos. When taking into account differing sample sizes there is no difference in diversity between the species (permutation test, test statistic = 3, one tailed p = 0.999). At the subspecies level, diversity levels also do not differ among any chimpanzee subspecies and bonobos (permutation test, observed test statistic = 0.070, p = 0.898). Identical PRDM9 DNA sequences were generally not shared between individuals of different subspecies or species, with one exception: A DNA sequence (p6), that we identified in four central chimpanzee individuals, was also reported in an eastern chimpanzee [36]. Overall, the combined data set consists of five unique DNA sequences found in the eastern chimpanzee sample, four in the central chimpanzees, 16 in the western chimpanzee sample and a further three in the bonobos, amounting to 27 unique DNA sequences, since one is shared among eastern and central chimpanzees (p6 = E1, Table 1). The p1 (A1) and p11 (B2) sequences differ only by two synonymous substitutions and thus the bonobo and eastern chimpanzee samples share an allele at the amino acid level, which has been described as a putatively ancestral allele [36]. The published PRDM9 sequence (GU166820, [19]) from a chimpanzee of unknown subspecies affiliation represents an additional unique allele not found in our sample.

**Table 1:** Distribution of alleles according to subspecies/species.

Species	Alleles (nt/AA)	n	Alleles nt (# of occurrences)	Alleles AA (# of occurrences)
*P. t. schweinfurthii*	5/5	10	p1 = A1(6), p2(1), p3(1), p4(1), E1*(1)	P1 = A1(6), P2(1), P3(1), P4(1), E1*(1)
*P. t. troglodytes*	4/4	7	p5(1), p6*(4), p7(1), p8(1)	P5(1), P6*(4), P7(1), P8(1)
*P. t. verus*	16/15	52	p9 = W1(12), p10(1), W2(1), W3a(2), W3b(2), W4(1), W5(3), W6(12), W7(1), W8(2), W9a(1), W9b(1), W10(1), W11a(1), W11b(7), W11c(4)	P9 = W1(12), P10(1), W2(1), W3a(2), W3b(2), W4(1), W5(3), W6(12), W7(1), W8(2), W9a(1), W9b(1), W10(1), W11a/b(8), W11c(4)
*P. paniscus*	3/3	12	p11 = B2(8), p12(2), B1(2)	P1 = B2(8), P11(2), B1(2)

Alleles (p1 = A1 and p11 = B2) differ only by two synonymous substitutions, so that bonobos and eastern chimpanzees share an allele (P1) at the amino acid level. Two alleles (W11a, W11b) identified in western chimpanzees differ only by one synonymous substitution and represent one allele at the amino acid level.

* There is one shared allele between central and eastern chimpanzees: alleles p6 and E1 are identical at the nucleotide level.

### Diversity in Chimpanzees in Comparison to Diversity in Humans

In order to compare the diversity observed to date in *Pan* to that reported in humans, we compiled the DNA sequences reported in four different studies of humans [6], [19], [24], [26]. We differentiated between DNA sequences found in individuals of mainly African and mainly non-African ancestry, but also analyzed the combined data, so that there were three data sets (Table S2). The resulting set of 446 human PRDM9 DNA sequences from individuals of mainly non-African ancestry contains 21 alleles, whereas the set comprised of individuals of mainly African descent consists of 134 DNA sequences containing 19 alleles. In total, the set comprising all humans regardless of their ancestry consists of 580 DNA sequences, containing 36 alleles. In contrast, 24 alleles were observed in the sample of 69 chimpanzee PRDM9 sequences. This suggests that the sample of chimpanzees harbors significantly more diversity at PRDM9 than the samples of non-African humans, African humans and humans in general (permutation test, test-statistic = 24, non-Africans: one tailed p = 0, Africans: one tailed p = 0, combined dataset: one tailed p = 0).

**Figure 3:**
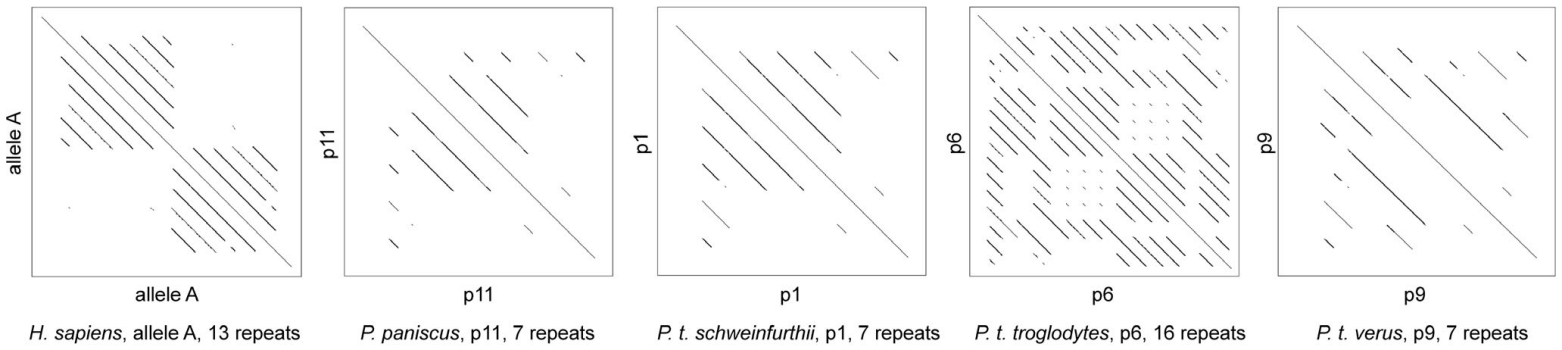
Self-comparison of predominant PRDM9 alleles. These diagrams depict the results of an analysis comparing PRDM9 DNA sequences to themselves with a window size of 83 and a mismatch limit of five. The main diagonal represents the alignment of a sequence to itself. The off-diagonal lines represent similar patterns within the sequences. The human allele shows a clear two-block structure, in which the repeats of the first half of the sequence are more similar to one another than to those in the second half of the sequence and vice versa. This structure is not seen in any of the *Pan* alleles.

It has been proposed that for human PRDM9, not all ZnFs present in the C-terminal array actually play a role in DNA binding. The repeats that are involved in the recognition of the 13 mer hotspot motif identified by Myers et al [21] are found in the C-terminal half of the ZnF array [6], [7], [24]. In humans, these ZnF repeats in the second half of the array are much more similar to one another than to any of the repeats in the first half of the array, and vice-versa. This is illustrated by the comparison of a sequence to itself in a dot-plot (Fig. 3). This type of structure in self-similarity was not found in the alleles we identified in *Pan* (Fig. 3).

### Testing for Signals of Positive Selection

The residues −1, 2, 3 and 6 of the alpha helix of each ZnF are of functional importance because they determine the DNA-binding specificity of the protein (e.g. [37]). A subset of these positions (−1, 3, and 6) have a high degree of variability and show strong signals of positive selection in rodents, as well as across the primate lineage [19], [20]. We examined whether the variation found in *Pan* PRDM9 ZnFs is consistent with a history of positive selection. This was done by first assessing a series of models, which either do or do not allow for positive selection (d_N_/d_S_ ratio (ω) >1) across the whole alignment.

All models that allow for positive selection also suggested positive selection in the data set and were favored over models not allowing for positive selection. For example, models M2a (selection) and M3 (discrete) both suggest that roughly 12.5% of sites are under strong positive selection with maximum likelihood estimates of ω = 8.28 and 8.08, respectively. Models M2a, M3 and M8 (beta+ω) all show significantly higher log likelihood values than the corresponding nested models (M0 vs M3∶2Δl = 29.61, df = 4, p = 0.00001; M1a vs M2a: 2Δl = 13.50, df = 2, p = 0.001; M7 vs M8∶2Δl = 14.33, df = 2, p = 0.0008).

Given the results suggesting a history of positive selection in the ZnF sequences, we next used a Bayes empirical Bayes (BEB) approach [38] to try and identify sites showing signs of positive selection. Under both models M2a and M8, the BEB analysis identified the same three amino acid sites as showing signs of positive selection (M2a: position −1: posterior probability P = 1.000**; posterior mean of ω = 8.017±1.736; pos 3: P = 0.964*, ω = 7.738±2.150; pos 6: P = 0.853, ω = 6.922±2.957 M8: position −1: P = 1.000** ω = 7.832±1.779 (SE); pos 3: P = 0.983*, ω = 7.699±1.982; pos 6: P = 0.905, ω = 7.118±2.661), which correspond to residues responsible for DNA binding specificity of the ZnF. We furthermore employed a sitewise likelihood-ratio method, which is a direct test for the location of selection [39] to confirm the results obtained by the previous approach. The SLR test detects positive selection at the same three residues under the F3×4 model and the incorporation of codon frequencies into the substitution matrix according to Muse and Gaut [40] with a p-value ≤0.05 (pos −1: ω = 18.630, p = 1.6373e−8, pos 3: ω = 8.976. p = 1.8045e−4, pos 6: ω = 5.186, p = 1.3003e−2; pos −1 and 3 are also significant after multiple testing correction at p≤0.01) When assuming a F61/F60 codon model, pos 6 is no longer identified as positively selected, while changing how codon frequencies are incorporated into the substitution matrix to the method described by Goldman and Whelan [41] leads to site 1 being additionally identified as having experienced positive selection. Overall, these analyses strongly suggest that the residues responsible for DNA binding specificity in *Pan* PRDM9 have been under strong positive selection, as has been previously demonstrated for the whole primate lineage and among rodents [19], [20].

## Discussion

In this study we assess DNA sequence diversity at PRDM9 in three subspecies of chimpanzees as well as bonobos. We find high levels of diversity in *Pan*, with 12 DNA sequences identified from a total of 22 individuals. When we analyze our data together with that of a recently published study, we find that one PRDM9 sequence is shared between members of the central and eastern chimpanzee subspecies. Although chimpanzees and bonobos do not share alleles at the DNA sequence level, two alleles identified in bonobos and eastern chimpanzees only differ by two synonymous substitutions so that they share an allele at the amino acid level. By finding no sharing of DNA sequences and only limited sharing of amino acid sequences between species, our results contrast with a recent study of autosomal non-coding regions of the genus *Pan* which demonstrated that bonobos fell within the variation of chimpanzees for many of the loci studied [42]. Although comparisons of diversity at noncoding, neutrally-evolving loci sequenced in bonobos as well as chimpanzees generally find levels of diversity in bonobos similar to that in any one chimpanzee subspecies [43], we found similar levels of variation at PRDM9 in chimpanzees and bonobos. In comparison to what we observed in chimpanzees, PRDM9 variation in humans appears more limited. We found a significant difference in PRDM9 variation between humans and chimpanzees when pooling all available human data, as well as when only considering humans of mainly non-African or mainly African ancestry. This is largely in agreement with general patterns of human autosomal genetic variation at non-coding loci and with current knowledge of relative levels of chimpanzee and human genetic diversity [43].

We also detected signals of positive selection in *Pan* PRDM9. The sites identified as having experienced positive selection are known to be contact residues, responsible for site-specific recognition. Our results are consistent with previous findings, which demonstrated positive selection on contact residues in rodents and primates, including humans [19], [20]. In sum, our findings suggest a different mode of evolution at PRDM9 than at neutral loci in *Pan*, raising the question of whether PRDM9 plays a similar role in hotspot recruitment in chimpanzees as it does in humans.

To explore this, we noted that the ZnFs of the second half of the human PRDM9 ZnF array are more similar to one another than to any of the repeats in the first half of the array, and vice-versa. This structure was not detected in any *Pan* PRDM9 ZnF array. In humans, only the ZnFs of the second half of the array are predicted to bind to the core 13 mer motif [6], [7], [21], [24]. However, Berg et al. [26] found that alleles that only differ in ZnFs in the first half of the array apparently have differing effects in hotspot recruitment. This suggests that the whole ZnF array plays a role in hotspot recruitment in humans, irrespective of the “2-block” structure. Thus, the apparent lack of this type of structure in *Pan* does not in itself signify that the gene is not active in hotspot regulation in this taxon.

There are no shared PRDM9 sequences between human and *Pan*, nor even sharing of individual ZnF sequences. This would suggest that, if PRDM9 is active in hotspot recruitment in chimpanzees, it activates hotspots distinct from human hotspots, which is in agreement with the lack of conservation of recombination hotspot locations between humans and chimpanzees [44], [45]. Auton et al. [36] note that they did not identify predicted PRDM9 binding sites, simple DNA motifs or repeat elements that are consistently associated with chimpanzee hotspots. The authors present three alternative explanations regarding this lack of association: 1) loss of function of PRDM9 in chimpanzees 2) recent origin of high allelic variation in PRDM9 masking signals for single alleles 3) individual alleles in chimpanzees binding to a greater number of target sequences than do human alleles. Our data are consistent with Auton et al.’s characterization of high allelic variation in western chimpanzees, albeit do not aid in distinguishing among these hypotheses.

It is worth emphasizing that while we are confident of the validity of the data presented here, our results possibly represent an underestimate of the diversity present in *Pan*. As described in detail in the supplementary information (Methods S1, Table S1, Table S3), we were successful in obtaining sequences from only 22 individuals. Direct sequencing of PCR products from ten of these individuals suggested the presence of more than one allele, but after repeated cloning and sequencing of multiple clones both alleles were obtained from only three of the ten apparently heterozygous individuals. PRDM9 is a member of a large gene family, which originated in Metazoans, expanded in vertebrates and experienced further duplications in primates. The presence of closely related paralogs in the genome, such as PRDM7, which apparently arose by duplication in primates [18], impedes specific targeting of the correct gene in the initial PCR step. Moreover, the repetitive structure of the ZnF array seemingly favors processes that lead to recombinant molecules either produced by *in vitro* recombination during PCR and/or due to mismatch-repair of heteroduplex molecules during cloning in *E. coli* [46]–[51]. It is conceivable that both processes contributed to the artefacts observed in this study. Additionally, long single reads are required to obtain reliable sequence data for the alleles with large fragment sizes (e.g. an amplicon with 17 repeats spans 1422 bp), since internal primers cannot be employed due to the highly repetitive structure of the array. Our data may therefore be biased towards shorter alleles. However, the human data used for comparison [6], [19], [24], [26] may also be similarly biased due to non-random sampling and the methods employed (e.g. MVR-PCR).

Although PRDM9 DNA sequences were generally not shared between individuals of different subspecies or species, the central and eastern chimpanzee and the eastern chimpanzee and bonobo samples share an allele at the DNA sequence and the functional level (due to two synonymous substitutions), respectively. This is not fully in agreement with the hypothesis that species should be distinguishable by their PRDM9 alleles and, strictly taken, does not support the idea that PRDM9 plays a universal role in hybrid sterility. However, because the ranges of chimpanzees and bonobos do not overlap, it is not clear whether hybridization is indeed possible between representatives of these species, or if any resulting offspring are themselves fertile. Eventual characterization of allelic variation in additional species will aid in addressing questions regarding the role of PRDM9 in meiotic recombination as well as in mediating hybrid sterility.

## Methods

### Samples

We used a total of 22 samples of the genus *Pan,* including five bonobos (*Pan paniscus*), seven eastern chimpanzees (*Pan troglodytes schweinfurthii*), six central chimpanzees (*P. t. troglodytes*) and four western chimpanzees (*P. t. verus*) obtained from in-house collections of the Genetics department of the MPI-EVA under the responsibility of Svante Pääbo. No new samples were collected for the purpose of this study. DNA samples used were derived from pre-existing materials at the MPI-EVA. The original source and geographic origin of the samples used in this study are listed in Table S4. Primate samples were collected during the course of routine veterinary procedures, were collected in accordance with regulations of the relevant governing agencies, and are used here in accordance with agreements established with the relevant animal sanctuaries. Other results from the samples used here were already published in [42].

### DNA Amplification and Sequencing

We sequenced the C-terminal zinc finger array of the gene PRDM9, excluding the first zinc finger repeat (“0” in schematic representation of PRDM9 in Fig. 1). The second repeat (first repeat in the actual C-terminal zinc finger array) is truncated and does not contain the first cysteine residue, as is known from primates and rodents [19]. The ZnF array was amplified and sequenced using previously published primers, which had been used successfully in humans (Table 2). Long range PCR amplifications were carried out in 50 µl reactions containing a final concentration of 3 mM MgCl_2_, 0.5 mM dNTPs, 0.4 µM each primer, 7 or 9% DMSO, 1× Expand Long Range buffer and 3.5 U Expand Long Range enzyme mix per 50 µl reaction (Roche Applied Science, Mannheim, Germany). Cycling conditions were 2 min initial denaturation at 92°C, ten cycles of 10 s denaturation, 15 s annealing and 2 min elongation at 68°C followed by 30 cycles during which the elongation time was increased by 20 s per cycle and a final elongation step of 7 min. Annealing temperatures were optimized as specified in Table 2. PCR products were excised from TAE gels and purified using QIAquick MinElute spin columns (Qiagen). Due to difficulties in obtaining unambiguous full length sequences from direct sequencing from all products, probably due to multiple polymorphic sites and length polymorphisms in heterozygous individuals, PCR products were cloned using a TOPO TA Cloning kit (Invitrogen, pCR2.1-TOPO vector, TOP10 chemically competent One Shot cells) to identify single alleles. For consistency, we also cloned and sequenced apparently homozygous products. Plasmid DNA was isolated using the PureLink Quick Plasmid Miniprep Kit (Invitrogen) via centrifugation. Both strands were sequenced using the primers listed in Table 2 employing the BigDye Terminator v3.1 Cycle Sequencing Kit (Applied Biosystems) on a 3730 DNA Analyzer (Applied Biosystems). The number of sequenced clones per individual ranged from five to 24 (average = 13, see Table S3 for details). All DNA sequences in our final data set were observed in at least four clones from two independent initial PCRs or in five clones from one initial PCR (Table S3). DNA sequences have been deposited at GenBank (Accession numbers: JQ771765–JQ771776).

**Table 2:** Primers used in this study.

Primer	Sequence 5′–3′	Reference	T_a_ °C
HsPrdm9_F3	TGTAAGGAATGACACTGCCCTGA	[6]	60
HsPrdm9_R1	ATGTCCCCCGAACACTTACAGAA	[6]	
PN0.6F	TGAGGTTACCTAGTCTGGCA	[24]	57
PN2.5R	ATAAGGGGTCAGCAGACTTC	[24]	
11F	GGACTGTAAAGGTCCATCCAGCACTTGG	[32]	68
11R	AAAGAACCACACATGCTGATGTCC	[32]	
11FS*	CATACCTTCATATGTGGTAAGGCC	[32]	
11RS*	TATAAGGGGTCAGCAGACTTCCGC	[32]	
11S1*	AAAGTCAAGTATGGAGAGTGTGG	[32]	

T_a_ °C =  annealing temperature in degrees Celsius.

* indicates primers used for sequencing.

### Sequence Data Analysis

Raw sequences were edited using CodonCodeAligner v3.7.1 (CodonCode Corporation, Dedham, MA, USA) and checked by eye. Subsequently, sequences were manipulated using SeaView v4.2.8 [52] and Se-Al v2.0a11 (Andrew Rambaut, http://tree.bio.ed.ac.uk/software/seal/) and collapsed into unique alleles using FaBox [53].

Dot plots of the highest frequency *Pan* PRDM9 alleles and the previously published human allele A were generated with a window size of 83 and a mismatch limit of five using a web-based tool (http://www.vivo.colostate.edu/molkit/dnadot/).

To test whether the observed differences in the number of unique alleles found within a species/subspecies was significant, permutation tests were carried out [54], [55]. 10,000 permutations were performed for the comparison among the three subspecies and bonobos, as well as the comparison between chimpanzees and bonobos, and chimpanzees and humans. Alleles were permuted over species/subspecies. The test statistic for the comparison between two groups (in this case species: human vs. chimpanzee and chimpanzee vs. bonobo) was the number of unique alleles in the smaller group. For the comparison among more than two groups (subspecies) the test statistic was the sum of squared deviations from the mean of the number of unique alleles per number of total alleles per group. The P values of the test statistics were the proportion of permutations that revealed a test statistic at least as large as that of the respective original data set. The comparisons between chimpanzees and bonobos, as well as among the chimpanzee subspecies and bonobos, were based on the DNA sequence data presented in this study plus data taken from Auton et al. [36] (Table 1). For the comparison with humans, data from four separate studies were compiled [6], [19], [24], [26] and aligned and alignments subsequently collapsed into unique alleles using using FaBox [53]. The first data set consisted of 21 unique alleles found among 446 individuals of mainly non-African ancestry and the second data set of 19 unique alleles found in a sample of 134 individuals of mainly African ancestry (Table S2). All permutation tests were conducted in R (R 2.11.1 GUI 1.34).

To detect sites under positive selection we used an alignment of all *Pan* ZnFs identified in our study, excluding the slightly aberrant first repeat. We employed both the method described by Nielsen and Yang [56] and Yang et al. [57] as implemented in codeml of the PAML package v4.4b [58], as well as the sitewise likelihood-ratio method using SLR v1.3 [39]. The former method takes information from all sites of the alignment into account, to estimate parameters that are common to all sites, in order to to identify whether the presence of positive selection can be inferred in general. Multiple nested pairs of models are assumed, which can then be compared by likelihood-ratio tests (LRT). If positive selection is detected by LRT in general, the location of sites under positive selection can be assessed through a post hoc Bayesian analysis (Bayes empirical Bayes  =  BEB) in a subsequent step [56], [57], [59]. We compared models M0 vs M3, M1a vs M2a, and M7 vs M8 [58]. The SLR method is a direct test for the location of selection. It tests each site for neutrality, while also estimating the parameters common to all sites based on the entire alignment. This method has been described to be less prone to type I errors than the former method and was used to confirm the results previously obtained using codeml [39]. We constrained the calculations to positively selected sites only and left all other parameters not mentioned here at their default value. To guard against potential convergence problems, each model/test was run twice and results were compared. Since tree topology (codeml, slr) and choice of codon model (codeml) did not influence the overall results, only the results from the best tree and the F3×4 model are presented for the codeml analyses. For the SLR tests, results from both codon models (F3×4, F61/F60) and both methods of codon frequency incorporation (freqtype: 1 and 2) are given.

The tree topology was estimated under maximum likelihood as implemented in Garli v2.0.1019 [60]. Two codon models were assumed. Both models calculate the codon frequencies as the product of the frequencies of the three nucleotides that constitute each codon as observed in the data. In the F3×4 model the nucleotide frequencies are based on each codon position separately, whereas the F1×4 model uses the nucleotide frequencies across all codon positions. The relative nucleotide rate parameters assumed by the codon model were set to the standard Goldman and Yang [61] model, with different substitution rates for transitions and transversions. Ten repetitions were carried out to verify consistency in log likelihood scores and obtained tree topologies. All other settings were left at their default value. Both codon models resulted in the same two tree topologies (F3×4: best tree 4×, 2nd tree 6×; F1×4: best tree 6×, 2nd tree 4×), which were used in the analyses of positive selection.

## Supporting Information

Table S1 **Expected and identified alleles of 22** ***Pan*** **samples.**
(DOC)

Table S2 **Compiled human PRDM9 alleles from four publications.**
(DOC)

Table S3 **Cloning results.**
(DOC)

Table S4 **Original source and geographic origin of samples included in the study.**
(DOC)

Methods S1 **Material and Methods.**
(DOC)

## References

[1] Lichten M, Goldman AS (1995). Meiotic recombination hotspots.. Annu Rev Genet.

[2] Petes TD (2001). Meiotic recombination hot spots and cold spots.. Nat Rev Genet.

[3] Arnheim N, Calabrese P, Tiemann-Boege I (2007). Mammalian Meiotic Recombination Hot Spots.. Annu Rev Genet.

[4] Coop G, Przeworski M (2007). An evolutionary view of human recombination.. Nat Rev Genet.

[5] Parvanov ED, Petkov PM, Paigen K (2010). Prdm9 controls activation of mammalian recombination hotspots.. Science.

[6] Baudat F, Buard J, Grey C, Fledel-Alon A, Ober C (2010). PRDM9 Is a Major Determinant of Meiotic Recombination Hotspots in Humans and Mice.. Science.

[7] Myers S, Bowden R, Tumian A, Bontrop RE, Freeman C (2010). Drive Against Hotspot Motifs in Primates Implicates the PRDM9 Gene in Meiotic Recombination.. Science.

[8] Mihola O, Trachtulec Z, Vlcek C, Schimenti JC, Forejt J (2009). A Mouse Speciation Gene Encodes a Meiotic Histone H3 Methyltransferase.. Science.

[9] Cheung VG, Sherman SL, Feingold E (2010). Genetics. Genetic control of hotspots.. Science.

[10] Hochwagen A, Marais GAB (2010). Meiosis: A PRDM9 Guide to the Hotspots of Recombination.. Curr Biol.

[11] McVean G, Myers S (2010). PRDM9 marks the spot.. Nat Genet.

[12] Goodstadt L, Ponting CP (2011). Is the control of recombination conserved among diverse eukaryotes?. Heredity.

[13] Sandovici I, Sapienza C (2010). PRDM9 sticks its zinc fingers into recombination hotspots and between species.. F1000 Biol Rep 2.

[14] Ponting CP (2011). What are the genomic drivers of the rapid evolution of PRDM9?. Trends Genet.

[15] Ségurel L, Leffler EM, Przeworski M (2011). The Case of the Fickle Fingers: How the PRDM9 Zinc Finger Protein Specifies Meiotic Recombination Hotspots in Humans.. PLoS Biol.

[16] Hayashi K, Yoshida K, Matsui Y (2005). A histone H3 methyltransferase controls epigenetic events required for meiotic prophase.. Nature.

[17] Birtle Z, Ponting CP (2006). Meisetz and the birth of the KRAB motif.. Bioinformatics.

[18] Fumasoni I, Meani N, Rambaldi D, Scafetta G, Alcalay M (2007). Family expansion and gene rearrangements contributed to the functional specialization of PRDM genes in vertebrates.. BMC Evol Biol.

[19] Oliver PL, Goodstadt L, Bayes JJ, Birtle Z, Roach KC (2009). Accelerated evolution of the Prdm9 speciation gene across diverse metazoan taxa.. PLoS Genet.

[20] Thomas JH, Emerson RO, Shendure J (2009). Extraordinary molecular evolution in the PRDM9 fertility gene.. PLoS ONE.

[21] Myers S, Freeman C, Auton A, Donnelly P, McVean G (2008). A common sequence motif associated with recombination hot spots and genome instability in humans.. Nat Genet.

[22] Boulton A, Myers RS, Redfield RJ (1997). The hotspot conversion paradox and the evolution of meiotic recombination.. P Natl Acad Sci Usa.

[23] Jeffreys AJ, Neumann R (2009). The rise and fall of a human recombination hot spot.. Nat Genet.

[24] Berg IL, Neumann R, Lam K-WG, Sarbajna S, Odenthal-Hesse L (2010). PRDM9 variation strongly influences recombination hot-spot activity and meiotic instability in humans.. Nat Genet.

[25] Kong A, Thorleifsson G, Gudbjartsson DF, Masson G, Sigurdsson A (2010). Fine-scale recombination rate differences between sexes, populations and individuals.. Nature.

[26] Berg IL, Neumann R, Sarbajna S, Odenthal-Hesse L, Butler NJ (2011). Variants of the protein PRDM9 differentially regulate a set of human meiotic recombination hotspots highly active in African populations.. Proceedings of the National Academy of Sciences.

[27] Hinch AG, Tandon A, Patterson N, Song Y, Rohland N (2011). The landscape of recombination in African Americans.. Nature.

[28] Fledel-Alon A, Leffler EM, Guan Y, Stephens M, Coop G (2011). Variation in human recombination rates and its genetic determinants.. PLoS ONE.

[29] Forejt J, Iványi P (1975). Genetic studies on male sterility of hybrids between laboratory and wild mice (*Mus musculus* L.).. Genet Res.

[30] Good JM, Handel MA, Nachman MW (2008). Asymmetry and polymorphism of hybrid male sterility during the early stages of speciation in house mice.. Evolution.

[31] Forejt J (1996). Hybrid sterility in the mouse.. Trends Genet.

[32] Irie S, Tsujimura A, Miyagawa Y, Ueda T, Matsuoka Y (2009). Single-nucleotide polymorphisms of the PRDM9 (MEISETZ) gene in patients with nonobstructive azoospermia.. Journal of Andrology.

[33] Axelsson E, Webster MT, Ratnakumar A, The LUPA Consortium, Ponting CP (2012). Death of PRDM9 coincides with stabilization of the recombination landscape in the dog genome.. Genome Res.

[34] Muñoz-Fuentes V, Di Rienzo A, Vilà C (2011). Prdm9, a major determinant of meiotic recombination hotspots, is not functional in dogs and their wild relatives, wolves and coyotes.. PLoS ONE.

[35] Grey C, Barthès P, Chauveau-Le Friec G, Langa F, Baudat F (2011). Mouse PRDM9 DNA-Binding Specificity Determines Sites of Histone H3 Lysine 4 Trimethylation for Initiation of Meiotic Recombination.. PLoS Biol.

[36] Auton A, Fledel-Alon A, Pfeifer S, Venn O, Ségurel L (2012). A fine-scale chimpanzee genetic map from population sequencing.. Science.

[37] Pabo CO, Peisach E, Grant RA (2001). Design and selection of novel Cys2His2 zinc finger proteins.. Annu Rev Biochem.

[38] Yang Z, Wong WSW, Nielsen R (2005). Bayes empirical bayes inference of amino acid sites under positive selection.. Mol Biol Evol.

[39] Massingham T, Goldman N (2005). Detecting amino acid sites under positive selection and purifying selection.. Genetics.

[40] Muse SV, Gaut BS (1994). A likelihood approach for comparing synonymous and nonsynonymous nucleotide substitution rates, with application to the chloroplast genome.. Mol Biol Evol.

[41] Goldman N, Whelan S (2002). A novel use of equilibrium frequencies in models of sequence evolution.. Mol Biol Evol.

[42] Fischer A, Prüfer K, Good JM, Halbwax M, Wiebe V (2011). Bonobos Fall within the Genomic Variation of Chimpanzees.. PLoS ONE.

[43] Fischer A, Pollack J, Thalmann O, Nickel B, Pääbo S (2006). Demographic history and genetic differentiation in apes.. Curr Biol.

[44] Ptak SE, Hinds DA, Koehler K, Nickel B, Patil N (2005). Fine-scale recombination patterns differ between chimpanzees and humans.. Nat Genet.

[45] Winckler W, Myers SR, Richter DJ, Onofrio RC, McDonald GJ (2005). Comparison of fine-scale recombination rates in humans and chimpanzees.. Science.

[46] Ennis PD, Zemmour J, Salter RD, Parham P (1990). Rapid cloning of HLA-A,B cDNA by using the polymerase chain reaction: frequency and nature of errors produced in amplification.. P Natl Acad Sci Usa.

[47] Pääbo S, Irwin DM, Wilson AC (1990). DNA damage promotes jumping between templates during enzymatic amplification.. Journal of Biological Chemistry.

[48] L’Abbé D, Belmaaza A, Décary F, Chartrand P (1992). Elimination of heteroduplex artifacts when sequencing HLA genes amplified by polymerase chain reaction (PCR).. Immunogenetics.

[49] Odelberg SJ, Weiss RB, Hata A, White R (1995). Template-switching during DNA synthesis by *Thermus aquaticus* DNA polymerase I. Nucleic Acids Res.

[50] Longeri M, Zanotti M, Damiani G (2002). Recombinant DRB sequences produced by mismatch repair of heteroduplexes during cloning in *Escherichia coli*.. European Journal of Immunogenetics.

[51] Zylstra P, Rothenfluh HS, Weiller GF, Blanden RV, Steele EJ (1998). PCR amplification of murine immunoglobulin germline V genes: strategies for minimization of recombination artefacts.. Immunol Cell Biol.

[52] Gouy M, Guindon S, Gascuel O (2010). SeaView version 4: A multiplatform graphical user interface for sequence alignment and phylogenetic tree building.. Mol Biol Evol.

[53] Villesen P (2007). FaBox: an online toolbox for fasta sequences.. Mol Ecol Notes.

[54] Adams DC, Anthony CD (1996). Using randomization techniques to analyse behavioural data.. Anim Behav.

[55] Manly B (1997). Randomization, Bootstrap and Monte Carlo Methods in Biology. New York: Chapman & Hall.. pp.

[56] Nielsen R, Yang Z (1998). Likelihood models for detecting positively selected amino acid sites and applications to the HIV-1 envelope gene.. Genetics.

[57] Yang Z, Nielsen R, Goldman N, Pedersen AM (2000). Codon-substitution models for heterogeneous selection pressure at amino acid sites.. Genetics.

[58] Yang Z (2007). PAML 4: Phylogenetic Analysis by Maximum Likelihood.. Mol Biol Evol.

[59] Anisimova M, Bielawski J, Yang Z (2001). Accuracy and Power of the Likelihood Ratio Test in Detecting Adaptive Molecular Evolution.. Mol Biol Evol.

[60] Zwickl D (2006). Genetic algorithm approaches for the phylogenetic analysis of large biological sequence datasets under the maximum likelihood criterion. The University of Texas at Austin.. pp.

[61] Goldman N, Yang Z (1994). A codon-based model of nucleotide substitution for protein-coding DNA sequences.. Mol Biol Evol.

