# Supplementary material for: High Diversity at PRDM9 in Chimpanzees and Bonobos
Source: PLoS One. 2012 Jul 2;7(7):e39064. doi: 10.1371/journal.pone.0039064 (PMC3388066; doi:10.1371/journal.pone.0039064)
Supplement: Table S1 — Expected and identified alleles of 22 Pan samples. (DOC) [file pone.0039064.s001.doc]

**Table S1.** **Expected and identified alleles of 22 *Pan* samples.**

| Name | Species | # alleles identified | # alleles expected * | # repeats** | Allele, nt | Allele, AA | GenBank accession # |
| --- | --- | --- | --- | --- | --- | --- | --- |
| Becky | *P. t. schweinfurthii* | 1 | 1 | 7 | p1 | P1 | JQ771767 |
| Cindy | *P. t. schweinfurthii* | 1 | 1 | 7 | p1 | P1 | JQ771767 |
| Katie | *P. t. schweinfurthii* | 1 | 1 | 7 | p1 | P1 | JQ771767 |
| Kazahukire | *P. t. schweinfurthii* | 1 | 1 | 7 | p1 | P1 | JQ771767 |
| Kidogo | *P. t. schweinfurthii* | 1 | 2 | 17 | p2 | P2 | JQ771776 |
| Nakuu | *P. t. schweinfurthii* | 1 | 1 | 7 | p1 | P1 | JQ771767 |
| Sally | *P. t. schweinfurthii* | 2 | 2 | 14, 14 | p3, p4 | P3, P4 | JQ771772, JQ771773 |
| Agnagui | *P. t. troglodytes* | 1 | 1 | 7 | p5 | P5 | JQ771768 |
| Botsomi | *P. t. troglodytes* | 1 | 2 | 16 | p6 | P6 | JQ771774 |
| Fan Tuek | *P. t. troglodytes* | 2 | 2 | 14, 16 | p7, p6 | P7, P6 | JQ771771, JQ771774 |
| Gao | *P. t. troglodytes* | 1 | 2 | 16 | p6 | P6 | JQ771774 |
| Golfi | *P. t. troglodytes* | 1 | 2 | 12 | p8 | P8 | JQ771770 |
| Marcelle | *P. t. troglodytes* | 1 | 2 | 16 | p6 | P6 | JQ771774 |
| Agnetta | *P. t. verus* | 1 | 1 | 7 | p9 | P9 | JQ771766 |
| Louise | *P. t. verus* | 1 | 1 | 7 | p9 | P9 | JQ771766 |
| Oscar | *P. t. verus* | 1 | 1 | 7 | p9 | P9 | JQ771766 |
| Small Lucie | *P. t. verus* | 1 | 2 | 17 | p10 | P10 | JQ771775 |
| Likasi | *P. paniscus* | 1 | 1 | 7 | p11 | P1 | JQ771765 |
| Limbuko | *P. paniscus* | 1 | 2 | 7 | p11 | P1 | JQ771765 |
| Ludwig | *P. paniscus* | 1 | 1 | 8 | p12 | P11 | JQ771769 |
| Malou_L | *P. paniscus* | 1 | 1 | 7 | p11 | P1 | JQ771765 |
| Ulindi | *P. paniscus* | 2 | 2 | 7,8 | p11, p12 | P1, P11 | JQ771765, JQ771769 |
| GU166820 | *P. t. ssp.* | na | na | 15 | p13 | P12 | GU166820 |

The GenBank sequence GU166820 is listed for comparison. *according to direct sequencing of PCR products **in C-terminal array, excluding repeat directly after SET domain, but including first slightly aberrant repeat.
