# Supplementary material for: High Diversity at PRDM9 in Chimpanzees and Bonobos
Source: PLoS One. 2012 Jul 2;7(7):e39064. doi: 10.1371/journal.pone.0039064 (PMC3388066; doi:10.1371/journal.pone.0039064)
Supplement: Table S2 — Compiled human PRDM9 alleles from four publications. (DOC) [file pone.0039064.s002.doc]

**Table S2: Compiled human PRDM9 alleles from four publications** [1-4]**.**

| Allele | Oliver et al 2009 [1] | Baudat et al 2010 [2] | Berg et al 2010+2011 [3,4] | Berg et al 2010+2011 [3,4] | Absolute numbers | | Percentages | |
| --- | --- | --- | --- | --- | --- | --- | --- | --- |
|  | Non-African | Non-African | Non-African | African | African | Non-African | African | Non-African |
| L1 |  |  | 1 |  | 0 | 1 | 0.00 | 0.22 |
| L2 |  |  | 1 |  | 0 | 1 | 0.00 | 0.22 |
| L3 |  |  | 1 |  | 0 | 1 | 0.00 | 0.22 |
| L4 |  |  |  | 6 | 6 | 0 | 4.48 | 0.00 |
| L5 |  |  |  | 1 | 1 | 0 | 0.75 | 0.00 |
| L6 |  |  |  | 8 | 8 | 0 | 5.97 | 0.00 |
| L7 |  |  | 1 | 3 | 3 | 1 | 2.24 | 0.22 |
| L8 |  |  | 1 |  | 0 | 1 | 0.00 | 0.22 |
| L9 |  |  | 2 |  | 0 | 2 | 0.00 | 0.45 |
| L10 |  |  | 1 |  | 0 | 1 | 0.00 | 0.22 |
| L11 |  |  |  | 4 | 4 | 0 | 2.99 | 0.00 |
| L12 |  |  |  | 2 | 2 | 0 | 1.49 | 0.00 |
| L13 |  |  |  | 1 | 1 | 0 | 0.75 | 0.00 |
| L14 |  |  |  | 7 | 7 | 0 | 5.22 | 0.00 |
| L15 |  |  |  | 3 | 3 | 0 | 2.24 | 0.00 |
| L16 |  |  |  | 3 | 3 | 0 | 2.24 | 0.00 |
| L17 |  |  |  | 1 | 1 | 0 | 0.75 | 0.00 |
| L18 |  |  |  | 1 | 1 | 0 | 0.75 | 0.00 |
| L19 |  |  |  | 2 | 2 | 0 | 1.49 | 0.00 |
| L20 |  |  | 8 |  | 0 | 8 | 0.00 | 1.79 |
| L21 |  |  |  | 2 | 2 | 0 | 1.49 | 0.00 |
| L22 |  |  |  | 2 | 2 | 0 | 1.49 | 0.00 |
| L23 |  |  |  | 1 | 1 | 0 | 0.75 | 0.00 |
| L24 |  |  | 3 |  | 0 | 3 | 0.00 | 0.67 |
| L25 |  |  | 1 |  | 0 | 1 | 0.00 | 0.22 |
| L26 |  |  | 1 |  | 0 | 1 | 0.00 | 0.22 |
| L27 |  |  |  |  | 0 | 0 | 0.00 | 0.00 |
| A | 40 | 189 | 149 | 63 | 63 | 378 | 47.01 | 84.75 |
| B | 5 | 11 | 3 | 5 | 5 | 19 | 3.73 | 4.26 |
| C |  | 3 | 4 | 19 | 19 | 7 | 14.18 | 1.57 |
| D |  | 4 | 3 |  | 0 | 7 | 0.00 | 1.57 |
| E |  | 3 | 6 |  | 0 | 9 | 0.00 | 2.02 |
| F |  |  |  |  | 0 | 0 | 0.00 | 0.00 |
| I |  |  |  |  | 0 | 0 | 0.00 | 0.00 |
| H |  |  |  |  | 0 | 0 | 0.00 | 0.00 |
| isolate6a | 1 |  |  |  | 0 | 1 | 0.00 | 0.22 |
| isolate10 | 1 |  |  |  | 0 | 1 | 0.00 | 0.22 |
| isolate10b | 1 |  |  |  | 0 | 1 | 0.00 | 0.22 |
| isolate18a | 1 |  |  |  | 0 | 1 | 0.00 | 0.22 |
| isolate7 | 1 |  |  |  | 0 | 1 | 0.00 | 0.22 |
|  |  |  |  |  |  |  |  |  |
|  |  |  |  |  |  |  |  |  |
| total | 50 | 210 | 186 | 134 | 134 | 446 | 100 | 100 |
|  |  |  |  |  |  |  |  |  |
|  |  |  |  |  |  |  | 15 under 5% | 20 under 5% |
|  |  |  |  |  |  |  | in a total of 134 | in a total of 446 |

Alleles are categorized according to whether they were found in individuals of mainly African or mainly non-African ancestry.

1. Oliver PL, Goodstadt L, Bayes JJ, Birtle Z, Roach KC, et al. (2009) Accelerated evolution of the Prdm9 speciation gene across diverse metazoan taxa. PLoS Genet 5: e1000753. doi:10.1371/journal.pgen.1000753.

2. Baudat F, Buard J, Grey C, Fledel-Alon A, Ober C, et al. (2010) PRDM9 Is a Major Determinant of Meiotic Recombination Hotspots in Humans and Mice. Science 327: 836–840. doi:10.1126/science.1183439.

3. Berg IL, Neumann R, Lam K-WG, Sarbajna S, Odenthal-Hesse L, et al. (2010) PRDM9 variation strongly influences recombination hot-spot activity and meiotic instability in humans. Nat Genet 42: 859–863. doi:10.1038/ng.658.

4. Berg IL, Neumann R, Sarbajna S, Odenthal-Hesse L, Butler NJ, et al. (2011) Variants of the protein PRDM9 differentially regulate a set of human meiotic recombination hotspots highly active in African populations. Proceedings of the National Academy of Sciences 108: 12378–12383. doi:10.1073/pnas.1109531108.
