# Supplementary material for: High Diversity at PRDM9 in Chimpanzees and Bonobos
Source: PLoS One. 2012 Jul 2;7(7):e39064. doi: 10.1371/journal.pone.0039064 (PMC3388066; doi:10.1371/journal.pone.0039064)
Supplement: Table S3 — Cloning results. (DOC) [file pone.0039064.s003.doc]

**Table S3: Cloning results.**

| Name | Species | Direct sequencing | | Allele 1 | | | | Allele 2 | | | | Artefacts | | Total # clones |
| --- | --- | --- | --- | --- | --- | --- | --- | --- | --- | --- | --- | --- | --- | --- |
|  |  | Primers | Hetero-zygous | Repeats | # | # | #PCR  errors | Repeats | # | # | #PCR errors | Repeats | #clones |  |
|  |  |  |  |  | clones | PCRs |  |  | Clones | PCRs |  |  |  |  |
| Becky | *P. t. schweinfurthii* | PN, HS | no | 7 | 15 | 4 | 15 |  |  |  |  | 3,5 | 1,1 | 17 |
| Cindy, Uganda | *P. t. schweinfurthii* | 11FR | no | 7 | 13 | 3 | 5 |  |  |  |  | 3,4,4,4,5,6 | 1,1,1,1,1,1 | 19 |
| Katie | *P. t. schweinfurthii* | 11FR | no | 7 | 15 | 2 | 11 |  |  |  |  | 4 | 1 | 16 |
| Kazahukire | *P. t. schweinfurthii* | HS | no | 7 | 12 | 3 | 17 |  |  |  |  |  |  | 12 |
| Nakuu | *P. t. schweinfurthii* | PN, HS | no | 7 | 18 | 2 | 6 |  |  |  |  | 6,8 | 1,1 | 20 |
| Agnagui | *P. t. troglodytes* | PN, HS | no | 7 | 20 | 3 | 14 |  |  |  |  | 6,6,6,2 | 1,1,1,1 | 24 |
| Agnetta | *P. t. verus* | 11FR | no | 7 | 11 | 1 | 4 |  |  |  |  |  |  | 11 |
| Louise | *P. t. verus* | 11FR | no | 7 | 17 | 3 | 6 |  |  |  |  | 5 | 1 | 18 |
| Oscar | *P. t. verus* | 11FR | no | 7 | 7 | 1 | 9 |  |  |  |  | 6 | 1 | 8 |
| Likasi | *P. paniscus* | PN, 11FR | no | 7 | 12 | 2 | 14 |  |  |  |  | 2,6 | 1,1 | 14 |
| Ludwig | *P. paniscus* | HS | no | 8 | 7 | 1 | 2 |  |  |  |  |  |  | 7 |
| Malou_L | *P. paniscus* | 11FR | no | 7 | 15 | 3 | 11 |  |  |  |  | 3,5 | 1,1 | 17 |
| Sally | *P. t. schweinfurthii* | PN, 11FR | yes | 14 | 10 | 3 | 4 | 14 | 5 | 2 | 3 | 15 | 1 | 16 |
| Fan Tuek | *P. t. troglodytes* | 11FR | yes | 14 | 5 | 2 | 1 | 16 | 5 | 2 | 2 | 12, 12 | 1,1 | 12 |
| Ulindi | *P. paniscus* | HS | yes | 7 | 7 | 2 | 3 | 8 | 5 | 2 | 1 |  |  | 12 |
| Botsomi | *P. t. troglodytes* | 11FR | yes | 16 | 4 | 2 | 0 |  |  |  |  | 13,12,9 | 1,1,1 | 7 |
| Kidogo | *P. t. schweinfurthii* | 11FR | yes | 17 | 6 | 1 | 3 |  |  |  |  | 15 | 1 | 7 |
| Gao | *P. t. troglodytes* | 11FR | yes | 16 | 6 | 2 | 2 |  |  |  |  | 18,17,14,14,10,9 | 1,2,1,1,1,1 | 13 |
| Golfi | *P. t. troglodytes* | PN, 11FR | yes | 12 | 7 | 2 | 3 |  |  |  |  | 15,10 | 1,1 | 9 |
| Marcelle | *P. t. troglodytes* | 11FR | yes | 16 | 11 | 2 | 9 |  |  |  |  | 16,15,15,14,13 | 1,2,1,1,1 | 17 |
| Small Lucie | *P. t. verus* | 11FR | yes | 17 | 5 | 1 | 1 |  |  |  |  | 17,15,2 | 2,1,2 | 10 |
| Limbuko | *P. paniscus* | 11FR | yes | 7 | 13 | 3 | 8 |  |  |  |  | 8,6,4,4,4,3 | 3,1,1,1,1,1 | 21 |

Sequence data from the 22 *Pan* samples were included in the final data set. For some samples, two direct sequences stemming from different initial primer pairs are available (third column). “# PCR errors” refers to single nucleotide substitutions observed among all forward and reverse sequences of all clones per sample and allele. Abbreviations for primer pairs: HS= HsPrdm9_F3 and HsPrdm9_R1, PN= PN0.6F and PN2.5R, 11FR = 11F and 11R. Primer sequences and references are listed in Table 2.
