# Supplementary material for: High Diversity at PRDM9 in Chimpanzees and Bonobos
Source: PLoS One. 2012 Jul 2;7(7):e39064. doi: 10.1371/journal.pone.0039064 (PMC3388066; doi:10.1371/journal.pone.0039064)
Supplement: Table S4 — Original source and geographic origin of samples included in the study. (DOC) [file pone.0039064.s004.doc]

**Table S4: Original source and geographic origin of samples included in the study.**

| Name | Species | Source and geographic origin | Wildborn |
| --- | --- | --- | --- |
| Becky | *P. t. schweinfurthii* | Uganda, confiscated in Kampala in Jan 1994, since then Ngamba island | y |
| Cindy, Uganda | *P. t. schweinfurthii* | Budongo Forest, Uganda | y |
| Katie | *P. t. schweinfurthii* | DRC, confiscated Entebbe Airport (Uganda), Dec 1990, since then Ngamba island | y |
| Kazahukire | *P. t. schweinfurthii* | Uganda, confiscated in Hoima, 13 Aug. 2002, since then Ngamba island | y |
| Kidogo | *P. t. schweinfurthii* | DRC, confiscated in 1988, since then Ngamba island | y |
| Nakuu | *P. t. schweinfurthii* | DRC, confiscated in Kampala, 29 May 2002, since then Ngamba island | y |
| Sally | *P. t. schweinfurthii* | Uganda, confiscated in Kampala, Jan 1994, since then Ngamba island | y |
| Agnagui | *P. t. troglodytes* | Loukolela, Republic of the Congo, later Tchimpounga sanctuary | y |
| Botsomi | *P. t. troglodytes* | Republic of the Congo, later Tchimpounga sanctuary | y |
| Fan Tuek | *P. t. troglodytes* | Republic of the Congo, Zoo Brazzaville, later Tchimpounga sanctuary | y |
| Gao | *P. t. troglodytes* | Republic of the Congo, PNR, later Tchimpounga sanctuary | y |
| Golfi | *P. t. troglodytes* | Republic of the Congo, Zoo Brazzaville, later Tchimpounga sanctuary | y |
| Marcelle | *P. t. troglodytes* | na, later Tchimpounga sanctuary | y |
| Agnetta | *P. t. verus* | Biomedical Primate Research Center, Rijswijk, The Netherlands | n |
| Louise | *P. t. verus* | Biomedical Primate Research Center, Rijswijk, The Netherlands | n |
| Oscar | *P. t. verus* | Biomedical Primate Research Center, Rijswijk, The Netherlands | n |
| Small Lucie | *P. t. verus* | confiscated in Pujehun town, Pujehun district, Sierra Leone, later Tacugama | y |
| Likasi | *P. paniscus* | DRC, later Lola ya bonobo | y |
| Limbuko | *P. paniscus* | Zoo Leipzig (born in Stuttgart) | n |
| Ludwig | *P. paniscus* | Antwerp | n |
| Malou_L | *P. paniscus* | DRC, later Lola ya bonobo | y |
| Ulindi | *P. paniscus* | Zoo Leipzig (born in Frankfurt) | n |

na=data not available
