# Supplementary material for: High Diversity at PRDM9 in Chimpanzees and Bonobos
Source: PLoS One. 2012 Jul 2;7(7):e39064. doi: 10.1371/journal.pone.0039064 (PMC3388066; doi:10.1371/journal.pone.0039064)
Supplement: Methods S1 — Material and Methods. (DOC) [file pone.0039064.s005.doc]

**Methods S1. Material and Methods.**

Long range PCR amplifications did not always yield single products, but instead showed one bright band and multiple fainter bands of varying size on 0.8% TBE gels run for ~3hrs at 130V. Since the target size of products was expected to vary widely (for example the alleles that we identified varied from 7 to 17 terminal repeats; thus differing by 840bp in size), we chose the brightest band as our target.

In addition to the data reported here, we tested eight additional *Pan* samples, which either did not yield enough PCR product for downstream steps, failed completely in cloning, or produced non-alignable or non-reproducible sequences. The non-reproducible sequences appeared to be recombinant sequences; either produced by *in vitro* recombination during PCR or due to mismatch-repair of heteroduplex molecules during cloning [1-6]. For the individuals which produced reliable data, the total number of successfully sequenced clones per individual ranged from five to 24 (average = 13), whereas the final number of clones per allele, after exclusion of the cloning artefacts, ranged from four to 20. All DNA sequences in our final data set were observed in at least four clones from two independent initial PCRs or in five clones from one initial PCR (Table S3). In order to avoid the possibility of misidentifying potential artefacts (recombinant sequences) of an allele from this highly repetitive locus as a real allele, putative second alleles from individuals judged as apparently heterozygous according to the results of the direct sequencing were only accepted as real, if the DNA sequence was identified in at least five clones stemming from two independent PCRs.

1. Ennis PD, Zemmour J, Salter RD, Parham P (1990) Rapid cloning of HLA-A,B cDNA by using the polymerase chain reaction: frequency and nature of errors produced in amplification. P Natl Acad Sci Usa 87: 2833–2837.

2. Pääbo S, Irwin DM, Wilson AC (1990) DNA damage promotes jumping between templates during enzymatic amplification. Journal of Biological Chemistry 265: 4718.

3. L'Abbé D, Belmaaza A, Décary F, Chartrand P (1992) Elimination of heteroduplex artifacts when sequencing HLA genes amplified by polymerase chain reaction (PCR). Immunogenetics 35: 395–397.

4. Odelberg SJ, Weiss RB, Hata A, White R (1995) Template-switching during DNA synthesis by Thermus aquaticus DNA polymerase I. Nucleic Acids Res 23: 2049–2057.

5. Zylstra P, Rothenfluh HS, Weiller GF, Blanden RV, Steele EJ (1998) PCR amplification of murine immunoglobulin germline V genes: strategies for minimization of recombination artefacts. Immunol Cell Biol 76: 395–405. doi:10.1046/j.1440-1711.1998.00772.x.

6. Longeri M, Zanotti M, Damiani G (2002) Recombinant DRB sequences produced by mismatch repair of heteroduplexes during cloning in Escherichia coli. European Journal of Immunogenetics 29: 517–523.
